# Knowledge and Awareness Regarding Amblyopia Among Parents in Riyadh, Saudi Arabia: A Cross-Sectional Study

**DOI:** 10.7759/cureus.53308

**Published:** 2024-01-31

**Authors:** Othman J AlJarallah, Mohammed S AlFehaid, Aseel A Alnadawi, Saleh Ghulaysi, Alwaleed K Almouzan, Talal K Aljurayyan, Abdulaziz M Alnemari, Khalid Aldawsari, Hussam Almalki

**Affiliations:** 1 Surgery, Prince Sattam Bin Abdulaziz University, Al-Kharj, SAU; 2 College of Medicine, Prince Sattam Bin Abdulaziz University, Al-Kharj, SAU; 3 Medicine, Al Qassim University, Buraydah, SAU; 4 College of Medicine, Jazan University, Jazan, SAU; 5 Medicine and Surgery, College of Medicine, Umm Al-Qura University, Makkah, SAU

**Keywords:** amblyopia, riyadh, knowledge, poor vision, lazy eye

## Abstract

Introduction

Amblyopia is an abnormal development of usually one eye, leading to permanent decreased vision in the affected eye if not treated early. The condition is primarily caused by strabismus, cataract, anisometropic refractive error, or genetic factors and can manifest from birth to seven years of age, with a worldwide prevalence of 1.75%. This study examines parental knowledge of amblyopia in an effort to improve its early detection and intervention. Improving awareness can have a direct impact by lowering the incidence of untreated amblyopia and its related visual impairment. This study can also help healthcare professionals understand how to communicate with parents about amblyopia more effectively.

Materials and methods

This cross-sectional study included 417 parent participants from Riyadh, Saudi Arabia, selected through random sampling while ensuring representation from various governorates. The data was collected using an online questionnaire distributed by different social media platforms (Twitter, WhatsApp, and Telegram) to the parents of all governorates, and the sample was selected randomly. It was meticulously cleaned using Excel and analyzed with IBM Statistical Package for the Social Sciences (SPSS) version 29 (IBM Corp., Armonk, NY).

Results

Our results showed a predominant understanding of amblyopia as “poor vision in one or both eyes” (19.1%) as well as the role of eye movement or brain-eye coordination in the condition. Only 51.3% of parents were aware of lazy eye. Notably, the internet and doctors were the primary sources of information about amblyopia. Only 8.9% of the participants were aware that amblyopia cannot be treated after 10 years of age. Understanding the causes of amblyopia mainly included genetic factors and refractive errors. Sociodemographic factors such as gender, educational level, family history of eye disease, and having a child with a lazy eye significantly influenced the parents’ awareness levels of amblyopia.

Conclusion

Our study underscores the need for targeted educational initiatives to improve the knowledge and awareness of amblyopia among parents in Riyadh, Saudi Arabia. By addressing misconceptions, enhancing access to accurate information, and fostering a deeper understanding of amblyopia and its management, we can work toward ensuring timely diagnosis and appropriate interventions, ultimately reducing the prevalence and impact of amblyopia in the community.

## Introduction

Amblyopia, also referred to as “lazy eye,” is an abnormal development of usually one eye that causes the brain to ignore the visual signals from the affected eye and depend on the other eye. Over time, the vision of the weaker eye declines and worsens. Although amblyopia can happen in both eyes simultaneously, such cases are rare. Many conditions can be attributed to the development of amblyopia, the most common of which are strabismus, anisometropic refractive error, and cataracts. Amblyopia can happen from birth to seven years of age [1,2].

Amblyopia is the most common cause of visual acuity defects in children in developed countries, creating a burden both socially and economically [3]. Some studies have shown that the worldwide prevalence of amblyopia is 1.75% [4]. Estimates of its prevalence have varied in Saudi Arabia, including 1.3% in Jeddah [5], 3.9% in Al Qassim [6], and 1.85% in Abha [7]. However, a recent study reviewed seven papers from Saudi Arabia and estimated a prevalence of amblyopia of 2.3% [8].

Several cross-sectional studies have been conducted in various regions of Saudi Arabia to assess parental awareness of amblyopia. Two studies were conducted in Jeddah: Basheikh et al. surveyed 401 participants, with only 20% claiming sufficient knowledge about amblyopia, and Alzahrani et al. surveyed 474 participants, with a higher amblyopia awareness rate of 49.7% [9,10]. Almutairi et al. investigated amblyopia awareness in Hail, finding that 53.4% had heard of the condition [11]. Alsaqr et al. studied different regions of Saudi Arabia, revealing that only 30% of 1649 participants were familiar with the term “amblyopia” [12]. Surrati et al. conducted a study in Medina, where parental knowledge about children’s eye diseases varied among 773 participants, with 78.2% having poor awareness of the condition [13]. Alatawi et al. conducted a study in Tabuk, where 54.2% of 325 parents had never heard of amblyopia [14].

The above-mentioned studies collectively highlight varying levels of awareness across different regions in Saudi Arabia, indicating a need for targeted educational efforts to enhance knowledge about amblyopia. Amblyopia is a curable condition if diagnosed and treated early. Forcing the brain to use the affected eye by patching and limiting the use of the better eye is the gold standard of treatment. Lack of awareness and delayed diagnosis can lead to permanently reduced vision in the affected eye and even loss of vision. Children with amblyopia have a wide range of quality-of-life concerns as it affects their performance at school and everyday tasks such as driving, reading, and playing. Moreover, such children are at greater risk of depression, anxiety, and social difficulties [9]. If left untreated, amblyopia can result in permanent vision loss.

This study examines parental knowledge of amblyopia in an effort to improve early detection and intervention. By determining the level of parental knowledge that exists currently and identifying obstacles to early identification and treatment, it would be possible to create tailored educational initiatives and efforts to promote eye health. Improving the awareness of parents can have a direct impact on pediatric ophthalmology practice by lowering the incidence of untreated amblyopia and related visual impairment. This study can also help healthcare professionals understand how to communicate with parents more effectively, which will enhance patient awareness and medication compliance. To our knowledge, inadequate literature on the pertinent topic is available. Therefore, this study aims to increase awareness and knowledge of the condition by first assessing the awareness of amblyopia among parents in the Riyadh region of Saudi Arabia.

## Materials and methods

This work adopts an observational cross-sectional study design; the study was carried out from August 2023 to December 2023. The Riyadh region served as the study area, which included the Riyadh parent population. The Riyadh region, the capital of Saudi Arabia, includes more than 20 governorates with more than 8,500,000 people distributed among different governorates, towns, and villages. Ethical approval for the study was obtained from the ethical approval committee of Prince Sattam Bin Abdulaziz University (approval number SCBR-134/2023, on September 10, 2023). The researchers distributed a survey link via various social media platforms (Twitter, WhatsApp, and Telegram) to the parents of all governorates from towns and villages, and the sample was selected randomly. The inclusion criteria for the study were parents of children of either gender who were ≤18 years of age and who accepted to participate in the study. The exclusion criteria were parents of children who were >18 years of age and who refused to participate in the study.

The required sample sizes were calculated using the following equation:

n = z_1_^2^p (1p)/ d^2^

where n is the sample size, z = 1.96, and p is based on 50% of the value. As no previous research has been conducted in Riyadh to increase awareness and knowledge of amblyopia, we investigated the problem of amblyopia among men and women in the Riyadh region and obtained a sample size representative of the population there. Considering a desired marginal error of 0.05, a confidence level of 95%, and a non-response rate of 10%, a sample size of 417 participants was deemed appropriate, including both men and women. Parents of either male or female children in the Riyadh region were randomly selected. The data were collected using an electronic questionnaire divided into three parts. The first part included six questions about the demographics of the parents (i.e., age, gender, material status, education level, region of residence, and family history of amblyopia). The second part comprised seven questions regarding the parents' awareness of amblyopia and its diagnosis. The third part comprised four questions related to the parents' knowledge of amblyopia.

Descriptive statistics are used to describe the characteristics of the studied sample. The data were analyzed using IBM SPSS version 29 (IBM Corp., Armonk, NY) to generate descriptions of the demographic characteristics, scores of knowledge, and awareness about amblyopia. The variables were cross-tabulated, and their relationships were investigated according to variable type. The Fisher's exact test in addition to the chi-squared test was used when relevant. A p-value of <0.05 was considered statistically significant. The privacy of the participants was ensured as all questionnaires were anonymous, and no identifying data were collected. The data were stored in an electronic encrypted format and were only accessed by the investigators listed in the Google form application.

## Results

The study included 417 parents in Riyadh, Saudi Arabia, as shown in Table 1. The majority of the participants were female (73.9%) residing in Riyadh city (59.7%), with a significant portion aged 35-44 years (33.6%). Most had a monthly income of >10,000 SAR (64.0%) and a university education (71.2%). A notable proportion had a family history of eye disease (33.8%) or a child with lazy eye (9.6%).

**Table 1:** Sociodemographic and other parameters of the parents

Sociodemographic parameters		Frequency (n = 417)	Percent (%)
Gender	Female	308	73.9
	Male	109	26.1
Age (years)	<18	3	0.7
	18–24	66	15.8
	25–34	78	18.7
	35–44	140	33.6
	45–55	106	25.4
	>55	24	5.8
Residency	Riyadh city	249	59.7
	Riyadh governorates	168	40.3
Monthly income	<5,000 SAR	25	6.0
	5,000–10,000 SAR	125	30.0
	>10,000 SAR	267	64.0
Educational level	No education	8	1.9
	Primary education	8	1.9
	Secondary education	96	23.0
	Higher secondary education	8	1.9
	University education	297	71.2
Family history of eye disease	No	276	66.2
	Yes	141	33.8
Have a child with lazy eye	No	377	90.4
	Yes	40	9.6

**Figure 1:**
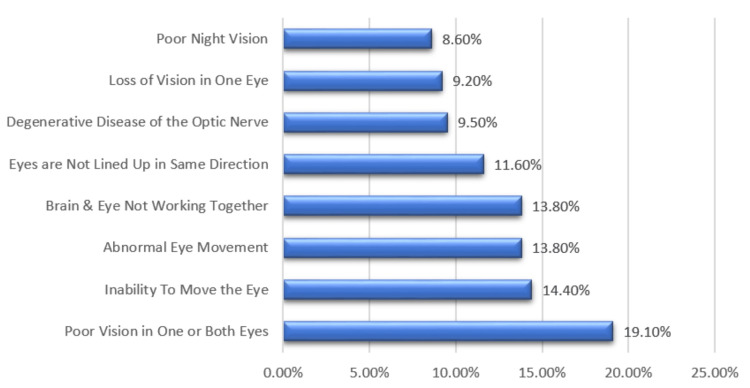
Parent responses when asked to define amblyopia

Figure 1 shows parents’ understanding of amblyopia among our study population. The majority associated it with “poor vision in one or both eyes” (19.1%); other misconceptions included “inability to move the eye” (14.4%) and “brain and eye not working together” (13.8%). Additional responses are shown in Figure 1.

**Figure 2:**
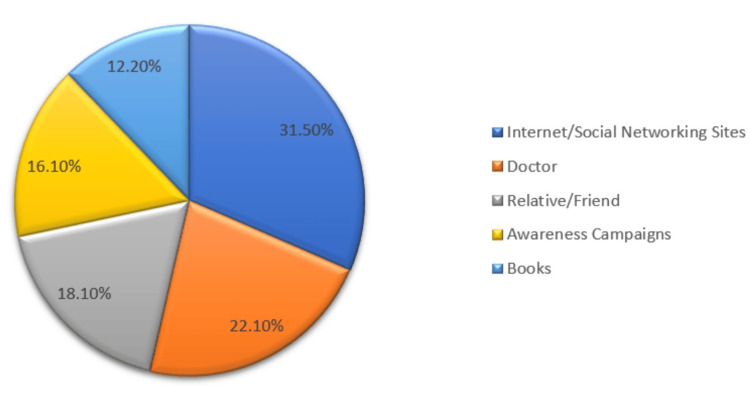
Parents’ sources of information about amblyopia

Figure 2 displays the sources from which parents in Riyadh, Saudi Arabia, gathered information about amblyopia. The internet and social networking sites were the primary sources (31.5%), followed by doctors (22.1%), relatives and friends (18.1%), awareness campaigns (16.1%), and books (12.2%).

Table 2 shows the data on amblyopia awareness among parents in our sample. About 51.3% had heard of the condition, whereas 38.1% had not, and 10.6% were unsure. Misconceptions of the condition existed among the participants, with some believing it could be diagnosed with the naked eye (20.6%) or by the patient themselves without visiting a specialist (11.8%). However, the majority correctly recognized that an ophthalmologist can diagnose it (84.2%). The majority (71.0%) believed all groups could be exposed to lazy eye. In relation to the treatment of lazy eye, 32.1% believed it could be treated after 10 years of age. Regarding the best age for treatment, 46.0% were unsure, but 91.1% emphasized the importance of checking a child’s vision before beginning school. Concerning the impact of untreated lazy eye, 77.7% acknowledged its negative effect on a child’s future.

**Table 2:** Knowledge and awareness of parents about amblyopia

Questions about knowledge/awareness	Responses	Frequency (n = 417)	Percent (%)
Ever heard of lazy eye	No	159	38.1
	Yes	214	51.3
	Don’t know	44	10.6
Lazy eye can be diagnosed with naked eye	No	116	27.8
	Yes	86	20.6
	Don’t know	215	51.6
Lazy eye can be diagnosed by patients with no need to visit a specialist doctor	No	368	88.2
	Yes	49	11.8
Lazy eye can be diagnosed by a pediatrician or family doctor	No	110	26.4
	Yes	151	36.2
	Don’t know	156	37.4
Lazy eye can be diagnosed by an ophthalmologist	No	8	1.9
	Yes	351	84.2
	Don’t know	58	13.9
Group exposed to lazy eye	Children	114	27.3
	Adults	7	1.7
	All of them	296	71.0
Lazy eye can be treated after 10 years of age	No	37	8.9
	Yes	134	32.1
	Don’t know	245	58.8
Best age to treat lazy eye	Don’t know	192	46.0
	No specific age	103	24.7
	Before 1 year	32	7.7
	3–9 years	75	18.0
	After 10 years	15	3.6
Important to check a child’s vision before beginning school	No	3	0.7
	Yes	380	91.1
	Don’t know	34	8.2
Lazy eye affects a child’s future if not treated	No	27	6.5
	Yes	324	77.7
	Don’t know	66	15.8

**Figure 3:**
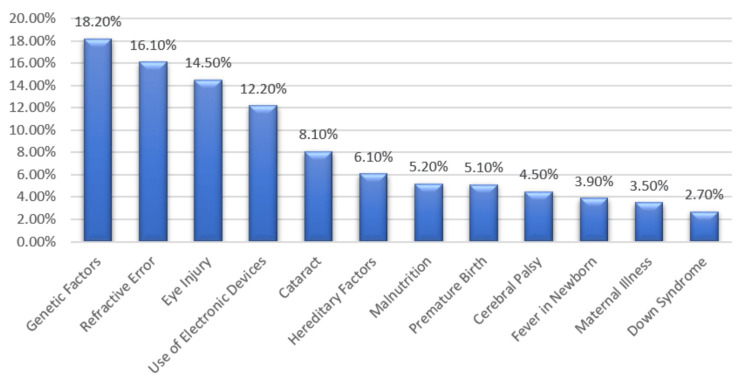
Knowledge about causes of amblyopia

Figure 3 shows the understanding of amblyopia’s causes among parents in our sample. Key factors identified were genetic influences (18.2%), refractive errors (16.1%), and eye injury (14.5%). Some misconceptions included the use of electronic devices (12.2%) and associations with cataracts (8.1%). Other less common but noted factors were hereditary factors, malnutrition, premature birth, cerebral palsy, and various medical conditions.

Table 3 shows the parents’ understanding of amblyopia treatment among our sample. The majority believed in the efficacy of eye muscle exercises (57.5%) and the prescription of glasses (48.4%) as potential treatments. Significant percentages also considered surgery (32.6%) and laser therapy (29.2%) to be effective. A notable portion believed in using a patch for the healthy eye (25.1%), whereas a minority thought no treatment was necessary (3.1%).

**Table 3:** Parents’ knowledge about the treatment of amblyopia

Questions assessing knowledge of amblyopia treatment	Frequency (n = 417)	Percent (%)
Exercises for eye muscles	240	57.5
Prescription of glasses	202	48.4
Surgery	136	32.6
Laser therapy	122	29.2
Patch for healthy eye	105	25.1
No treatment is required	13	3.1

Table 4 shows the association between various sociodemographic factors and parents’ awareness levels of amblyopia among our sample. The different age groups showed no significant difference in awareness levels. However, female parents exhibited higher awareness (p = 0.027). Family income and place of residence did not significantly impact awareness. Notably, a higher educational level corresponded to higher awareness. A significant association was found between a family history of eye disease and higher awareness (p < 0.001) as well as having a child with amblyopia (p < 0.001). Awareness levels significantly differed based on prior knowledge of amblyopia (p < 0.001).

**Table 4:** Associations between different sociodemographic factors and parents’ awareness of amblyopia P-value is calculated with Fisher's exact test, and p < 0.05 is significant.

Sociodemographic factors		Awareness level of amblyopia		p-value
		Poor awareness (n = 162)	High awareness (n = 255)	
Age of parent (years)	18–24	29	40	0.588
	25–34	33	45	
	35–44	52	88	
	45–55	42	64	
	>55	6	18	
Gender of parent	Female	110	198	0.027
	Male	52	57	
Family income	<5,000 SAR	12	13	0.557
	5,000–10,000 SAR	50	75	
	>10,000 SAR	100	167	
Residence area	Riyadh city	97	152	0.957
	Riyadh governorates	65	103	
Educational level	No education	3	5	0.776
	Primary education	3	5	
	Secondary education	42	54	
	Higher secondary education	2	6	
	University education	112	185	
Family history of eye disease	No	129	147	<0.001
	Yes	33	108	
Have a child with lazy eye	No	160	217	<0.001
	Yes	2	38	
Ever heard of lazy eye	No	98	61	<0.001
	Yes	36	178	
	Don’t know	28	16	

## Discussion

The study sought to assess the knowledge and awareness of amblyopia among parents in Riyadh, Saudi Arabia. The results shed light on various aspects of understanding, sources of information, awareness levels, causes, treatment perceptions, and the impact of sociodemographic factors on awareness. These findings provide valuable insights into the current state of amblyopia awareness and suggest potential areas for targeted education and intervention.

The findings revealed diverse levels of understanding regarding amblyopia among parents in Riyadh. A significant proportion correctly associated amblyopia with poor vision in one or both eyes [15]. Many parents associate the inability to move the eye or issues related to brain and eye coordination with amblyopia. Similarly, a study by Meier et al. showed that amblyopia has an adverse effect on the development of a binocular visual system and the interactions between signals from two eyes, indicating that amblyopia is associated with eye and brain coordination issues [16]. These findings point to a need for focused educational initiatives to ensure an accurate and comprehensive understanding of amblyopia among the general populace.

The study also highlighted the pivotal role of the internet and social networking sites as the primary sources of information on amblyopia, followed closely by medical practitioners, relatives, and awareness campaigns. Also, a study by Almutairi et al. showed that the most common sources of information were the Internet and social media [11]. These results emphasize the importance of utilizing various communication channels, especially digital platforms, to disseminate accurate and accessible information about amblyopia and its management.

Table 4 shows that the socio-demographic factors influenced parents' awareness levels regarding amblyopia, and the female parents exhibited a notably higher awareness compared to male parents. Additionally, a higher educational level was associated with greater awareness, underscoring the crucial role of education in fostering a deeper understanding of ocular health issues. Similarly, in a study by Bashir et al., most participants with a bachelor-level education represented the group with the highest awareness and knowledge about amblyopia [17]. Moreover, family history of eye disease and having a child with lazy eye were significantly associated with heightened awareness, emphasizing the role of personal experiences in shaping knowledge and awareness. Similarly, in a study by Alshaheen et al., awareness and perception levels of amblyopia were significantly higher among parents with a family history of amblyopia [18].

The findings regarding the understanding of amblyopia’s causes were notable, with genetic factors and refractive errors being the most widely recognized contributors [19,20]. However, our study also identified misconceptions related to electronic device usage and its association with the development of amblyopia. In contrast, Rodríguez-Abarca et al. showed in their study that electronic devices are useful in the management of amblyopia as symptoms improve with their usage [21]. These findings highlight the necessity of accurate and comprehensive information dissemination to address misconceptions and promote a better understanding of the multifactorial nature of amblyopia.

Regarding treatment, our study revealed various perceptions concerning the treatment of amblyopia, with a significant number of parents expressing belief in the effectiveness of eye muscle exercises and the prescription of glasses [22]. However, a considerable proportion of our participants also acknowledged the potential role of surgical interventions and laser therapy [23]. These results underscore the importance of providing comprehensive information about the range of available treatment options to ensure informed decision-making and promote early and effective intervention.

Based on the percentage of questions that were answered correctly by the participants, our study revealed a moderate level of awareness (51.3%) about lazy eye among parents in Riyadh, with over half of the participants being familiar with the condition. However, there still existed a notable percentage (49.7%) that were unaware or uncertain about key aspects of amblyopia, including its diagnosis and implications. These findings emphasize the need for targeted awareness campaigns and educational programs to bridge the existing gaps in knowledge and dispel misconceptions about amblyopia.

It is important to acknowledge certain limitations of our study. The sample primarily comprised parents living in Riyadh, Saudi Arabia, which may limit the generalizability of the findings to other regions or populations. Additionally, the study relied on self-reported data, which may be subject to recall bias or social-desirability bias. Further research involving larger and more diverse samples, including a more comprehensive assessment of knowledge and awareness levels, would provide a more nuanced understanding of amblyopia awareness among the broader population.

## Conclusions

Our study underscores the need for targeted educational initiatives to improve the knowledge and awareness of amblyopia among parents in Riyadh, Saudi Arabia. By addressing misconceptions, enhancing access to accurate information, and fostering a deeper understanding of amblyopia and its management, we can work toward ensuring timely diagnosis and appropriate interventions, ultimately reducing the prevalence and impact of amblyopia in the community.

## References

[1] (2022). Amblyopia (lazy eye). https://www.nei.nih.gov/learn-about-eye-health/eye-conditions-and-diseases/amblyopia-lazy-eye.

[2] (2021). Lazy eye (amblyopia). Mayo Clinic.

[3] Zagui RMB (2019). Amblyopia: types, diagnosis, treatment, and new perspectives. American Academy of Ophthalmology.

[4] Hashemi H, Fotouhi A, Yekta A, Pakzad R, Ostadimoghaddam H, Khabazkhoob M (2018). Global and regional estimates of prevalence of refractive errors: systematic review and meta-analysis. J Curr Ophthalmol.

[5] Bardisi WM, Bin Sadiq BM (2002). Vision screening of preschool children in Jeddah, Saudi Arabia. Saudi Med J.

[6] Aldebasi YH (2015). Prevalence of amblyopia in primary school children in Qassim province, Kingdom of Saudi Arabia. Middle East Afr J Ophthalmol.

[7] Abolfotouh MA, Badawi I, Faheem Y (1994). Prevalence of amblyopia among schoolboys in Abha city, Asir region, Saudi Arabia. J Egypt Public Health Assoc.

[8] Challa NK (2022). Prevalence of amblyopia among the children of Saudi Arabia: a systematic review, 1990-2020. African Vis Eye Health.

[9] Basheikh A, Alhibshi N, Bamakrid M, Baqais R, Basendwah M, Howldar S (2021). Knowledge and attitudes regarding amblyopia among parents in Jeddah, Saudi Arabia: a cross-sectional study. BMC Res Notes.

[10] Alzahrani N, Alhibshi N, Bukhari D (2018). Awareness, perceptions and knowledge of amblyopia among pediatrics and ophthalmology clinics attendees in King AbdulAziz University Hospital, Jeddah. Int J Adv Res.

[11] Almutairi MS, Alanezi NS, Alshammari FA (2022). Awareness and knowledge of amblyopia: a cross-sectional study among the population of Hail city, Saudi Arabia. Cureus.

[12] Alsaqr AM, Masmali AM (2019). The awareness of amblyopia among parents in Saudi Arabia. Ther Adv Ophthalmol.

[13] Surrati AM, Almuwarraee SM, Mohammad RA, Almatrafi SA, Murshid SA, Khayat LI, Al-Habboubi HF (2022). Parents’ awareness and perception of children’s eye diseases in Madinah, Saudi Arabia: a cross-sectional study. Cureus.

[14] Alatawi A, Alali N, Alamrani A, Hashem F, Alhemaidi S, Alreshidi S, Albalawi H (2021). Amblyopia and routine eye exam in children: parent's perspective. Children (Basel).

[15] Wong AM (2014). Amblyopia (lazy eye) in children. CMAJ.

[16] Meier K, Giaschi D (2017). Unilateral amblyopia affects two eyes: fellow eye deficits in amblyopia. Invest Ophthalmol Vis Sci.

[17] Bashir M, Melha AA, Alghamdi AF, Alc Hamad FH, Alzhrani AE, Embaby MM (2021). Level of awareness and perception of parents about amblyopia in children in Al-Baha city, Kingdom of Saudi Arabia (KSA). Medical Science.

[18] Alshaheen A, Owaifeer AA (2018). Amblyopia: parents' awareness and perceptions in Alhassa region of Saudi Arabia. Indo Am J Pharm Sci.

[19] Lee JY, Lee S, Park SK (2022). Genetic causal inference between amblyopia and perinatal factors. Sci Rep.

[20] Rajavi Z, Sabbaghi H, Baghini AS (2015). Prevalence of amblyopia and refractive errors among primary school children. J Ophthalmic Vis Res.

[21] Rodríguez-Abarca MA, Padilla-Alanís S, Rodríguez-Martínez AC, Mohamed-Noriega K, Fernández-de Luna ML (2022). Dichoptic binocular therapy and electronic devices for amblyopia treatment. Invest Ophthalmol Vis Sci.

[22] Rawstron JA, Burley CD, Elder MJ (2005). A systematic review of the applicability and efficacy of eye exercises. J Pediatr Ophthalmol Strabismus.

[23] Ajla P, Melisa AP, Ivana M, Senad G, Alma B, Aida P (2022). Laser keratomileusis in treatment of anisometropic amblyopia in adults. Taiwan J Ophthalmol.

